# The discovery of novel antitrypanosomal 4-phenyl-6-(pyridin-3-yl)pyrimidines

**DOI:** 10.1016/j.ejmech.2020.112871

**Published:** 2021-01-01

**Authors:** William J. Robinson, Annie E. Taylor, Solange Lauga-Cami, George W. Weaver, Randolph R.J. Arroo, Marcel Kaiser, Sheraz Gul, Maria Kuzikov, Bernhard Ellinger, Kuldip Singh, Tanja Schirmeister, Adolfo Botana, Chatchakorn Eurtivong, Avninder S. Bhambra

**Affiliations:** a Leicester School of Allied Health Sciences, De Montfort University, The Gateway, Leicester, LE1 9BH, UK; b Department of Chemistry, Loughborough University, Loughborough, LE11 3TU, UK; c Leicester School of Pharmacy, De Montfort University, The Gateway, Leicester, LE1 9BH, UK; d Swiss Tropical and Public Health Institute, Socinstrasse 57, 4051, Basel, Switzerland; e University of Basel, Petersplatz 1, 4003, Basel, Switzerland; f Fraunhofer Institute for Molecular Biology and Applied Ecology Screening Port, Hamburg, Germany; g Fraunhofer Cluster of Excellence Immune-Mediated Diseases CIMD, Hamburg Site, Hamburg, Germany; h Department of Chemistry, University of Leicester, Leicester, LE1 7RH, UK; i Institute of Pharmaceutical and Biomedical Sciences, Johannes Gutenberg University of Mainz, Staudingerweg 5, D-55128, Mainz, Germany; j JEOL UK, JEOL House, Silvert Court, Watchmead, Welwyn Garden City, Herts, AL7 1LT, UK; k Program in Chemical Sciences, Chulabhorn Graduate Institute, Chulabhorn Royal Academy, Bangkok, 10210, Thailand

**Keywords:** *Antitrypanosomal*, *Sleeping sickness*, *Human african trypanosomiasis*, *Trypanosoma brucei rhodesiense*, *Pyrimidines*, *Rhodesain*, *Docking*, *ADME-Tox*

## Abstract

Human African trypanosomiasis, or sleeping sickness, is a neglected tropical disease caused by *Trypanosoma brucei rhodesiense* and *Trypanosoma brucei gambiense* which seriously affects human health in Africa. Current therapies present limitations in their application, parasite resistance, or require further clinical investigation for wider use. Our work herein describes the design and syntheses of novel antitrypanosomal 4-phenyl-6-(pyridin-3-yl)pyrimidines, with compound **13**, the 4-(2-methoxyphenyl)-6-(pyridine-3-yl)pyrimidin-2-amine demonstrating an IC_50_ value of 0.38 μM and a promising off-target ADME-Tox profile *in vitro*. *In silico* molecular target investigations showed rhodesain to be a putative candidate, supported by STD and WaterLOGSY NMR experiments, however, *in vitro* evaluation of compound **13** against rhodesain exhibited low experimental inhibition. Therefore, our reported library of drug-like pyrimidines present promising scaffolds for further antikinetoplastid drug development for both phenotypic and target-based drug discovery.

## Highlights

• Human African trypanosomiasis (sleeping sickness) is a neglected tropical disease.
• Current drugs present limitations and severe side-effects in their application.
• We describe the syntheses of antitrypanosomal 4-phenyl-6-(pyridin-3-yl)pyrimidines.
• **13** showed potent antitrypanosomal activity *in vitro* against *T.b.r* (0.38 μM).

## Graphical abstract

**Figure:**
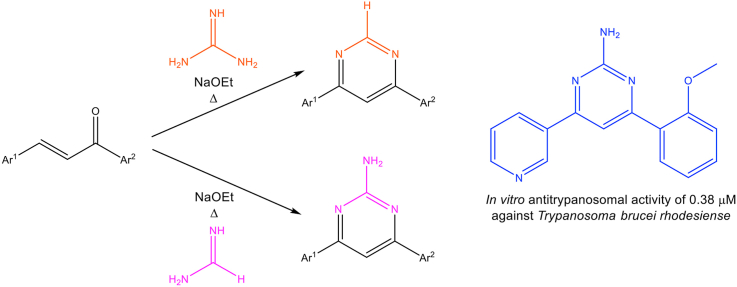

## 1 Introduction

Human African trypanosomiasis (HAT), also known as sleeping sickness, is a vector-borne parasitic disease caused by *Trypanosoma brucei rhodesiense* (*T.b.r*) and *Trypanosoma brucei gambiense* (*T.b.g*), two haemoflagellate subspecies of *Trypanosoma brucei* [1]. The disease is endemic in 36 sub-Saharan Africa countries and parasites are typically transmitted to humans by the tsetse fly. Infection with *T.b.r* manifests into the acute form of the disease whilst *T.b.g* causes the chronic form of illness. HAT has two notable stages where the first, known as the haemolymphatic stage occurs when trypomastigotes circulate in the bloodstream and lymphatic fluids. If untreated, the second stage, also known as the neurological stage ensues where parasites cross the blood brain barrier and patient recovery is unlikely leading to death.

**Fig. 1:**
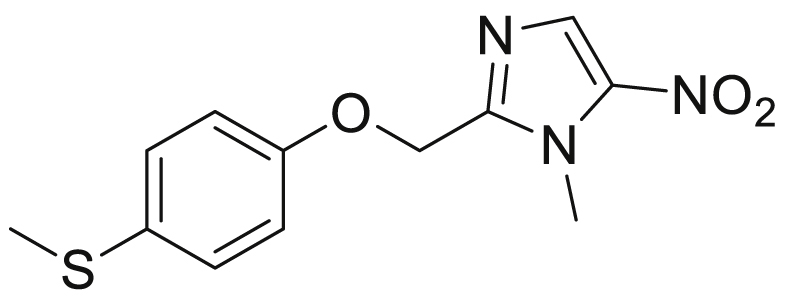
Fexinidazole.

No vaccines exist to treat sleeping sickness and chemotherapeutic treatments including pentamidine, melarsoprol, suramin and NECT have been associated with undesirable dosing regimens and unwanted side-effects including patient death [2,3]. Recently, fexinidazole (Fig. 1) was approved to treat infections caused by *T.b.g* specifically in the Democratic Republic of Congo (DRC) which is host to around 85% of cases recorded. However, factors affecting the success of fexinidazole will include the further success of drugs under evaluation for clinical and veterinary use, drug resistance and reservoir control of causative pathogens [[4], [5], [6]].

The use of privileged structures or scaffolds as building blocks in medicinal chemistry provide opportunities for developing new compound structures from an already active framework [7]. This strategy, combined with concepts such as scaffold hopping can support the generation of detailed structure activity relationships in a bid to develop suitable compounds for preclinical evaluation. For example, chalcones which contain the α, β-unsaturated carbonyl moiety form the core chemical scaffold for a range of natural products found within fruits, vegetables and other plants. This structural motif also presents in medications, including sofalcone which is used as a gastric mucosa protective agent [8,9].

Another important class of compounds accessible from chalcones are pyrimidines, which have been reported for a wide range of biological activities and are incorporated in clinically used drugs such as 5-fluorouracil [10]. Moreover, antitrypanosomal activities have been reported for compounds bearing the pyrimidine functional group demonstrating the significance of therapeutic potential [11,12]. Within this context, our previous work reported the syntheses of novel antitrypanosomal pyridylchalcones showing sub-micromolar IC_50_ values against *T.b.r* [13]. Using this chemical scaffold as a suitable template for modification, we investigated the syntheses and antitrypanosomal activities of substituted 4-phenyl-6-(pyridin-3-yl)pyrimidines in a continued interest in antikinetoplastid drug development [14].

## 2 Results and discussion

### 2.1 Syntheses

**Scheme 1:**
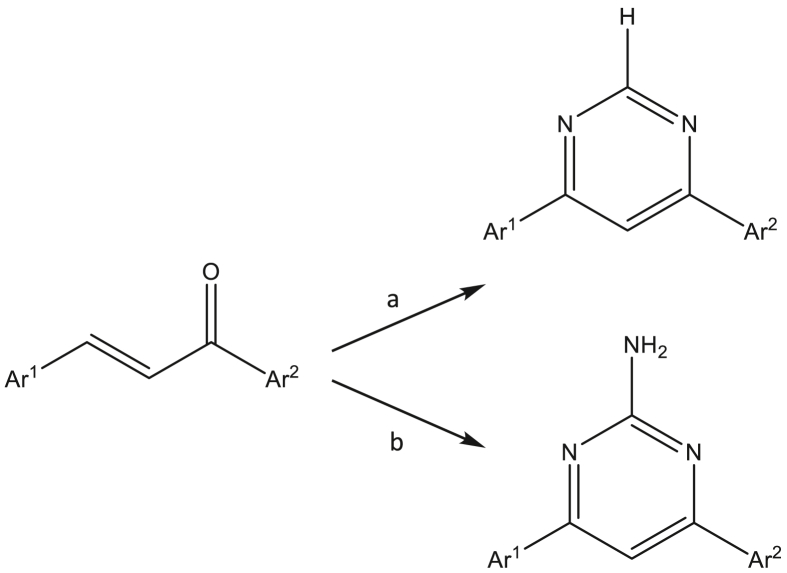
Reagents and conditions: a) sodium ethoxide, formamidine, reflux b) sodium ethoxide, guanidine hydrochloride, reflux.

Chalcones were synthesised as previously described by Bhambra et al. [13] and subsequently converted to corresponding 4-phenyl-6-(pyridin-3-yl)pyrimidines (Scheme 1) as reported by Varga et al. [15]. The synthesis was achieved in a one pot procedure by treating chalcones (1 mmol) with either formamidine or guanidine (1.5 mmol) and sodium ethoxide (4 mmol). Compounds were purified by recrystallisation or column chromatography using either ethyl acetate/hexane or dichloromethane/methanol as the eluent. The structures of new compounds were fully in accord with their analytical and spectroscopic properties.

**Fig. 2:**
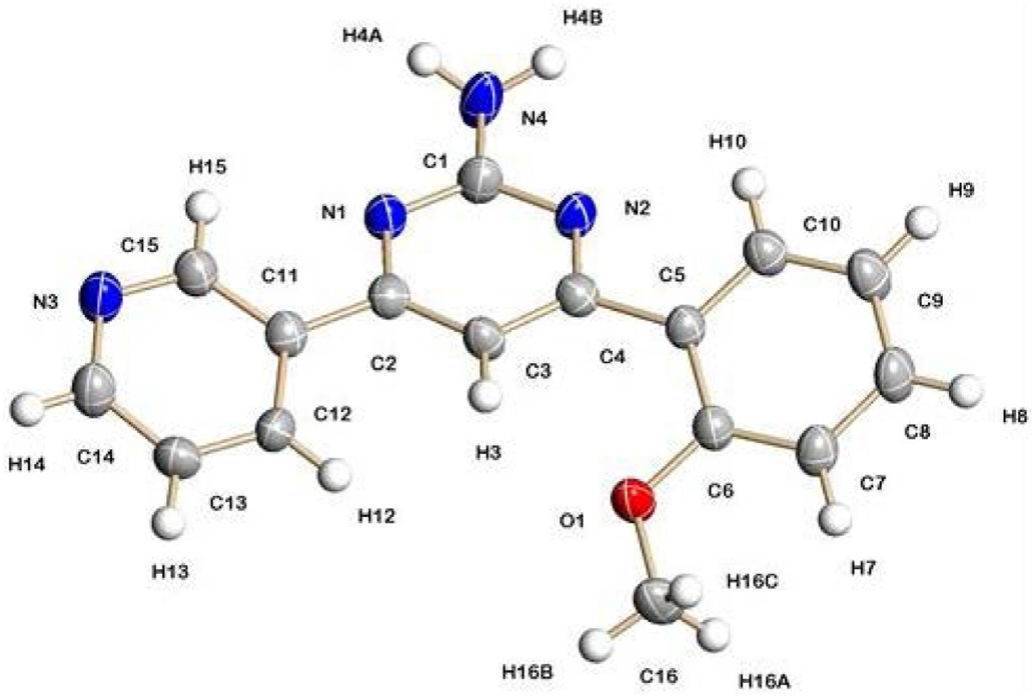
Crystal structure of compound **13**.

The structure of **13** was confirmed by single crystal X-ray diffraction analysis, with the molecular structure shown in Fig. 2.

### 2.2 Antitrypanosomal and antiproliferative activity

The antitrypanosomal activities (Table 1) of our 4-phenyl-6-(pyridin-3-yl)pyrimidines were determined against *Trypanosoma brucei rhodesiense* (STIB900) and cytotoxicity was assessed using L6 cells (rat skeletal myoblasts) as described by Bernal et al. [16].

**Table 1:** Antitrypanosomal and cytotoxic evaluation of synthesised novel 4-phenyl-6-(pyridin-3-yl)pyrimidines. SD – standard deviation.


Compound	Ar (A-ring)	Ar’ (B-ring)	R^1^	IC_50_ (μM)	±SD	CC_50_ (μM)	±SD	SI
				*T. b. rhodesiense*		L6 cells		
1	3-Py-	Ph-	H	4.8	0.21	>100	–	–
**2**	3-Py-	3-Pyridyl-	H	34.7	1.81	>100	–	–
**3**	3-Py-	2-OMe-Ph-	H	19.6	2.82	>100	–	–
**4**	3-Py-	2-Br-Ph-	H	13.5	2.58	>100	–	–
**5**	3-Py-	3-OMe-Ph-	H	8.0	1.13	>100	–	–
**6**	3-Py-	3-Br-Ph-	H	2.4	0.22	>65	–	–
**7**	3-Py-	3-F-Ph-	H	12.4	1.07	>100	–	–
**8**	3-Py-	4-OMe-Ph-	H	6.8	0.54	>100	–	–
**9**	3-Py-	3,4-OMe-	H	12.0	4.36	>100	–	–
**10**	3-Py-	3,4-O_2_CH_2_-Ph-	H	6.7	2.52	>100	–	–
**11**	3-Py-	Ph-	NH_2_	13.8	9.06	>100	–	–
**12**	3-Py-	3-Pyridyl-	NH_2_	57.4	3.40	>100	–	–
**13**	3-Py-	2-OMe-Ph-	NH_2_	0.38	0.18	23	7.38	61
**14**	3-Py-	2-Br-Ph-	NH_2_	20.2	4.71	>100	–	–
**15**	3-Py-	3-OMe-Ph-	NH_2_	12.0	0.36	>100	–	–
**16**	3-Py-	3-Br-Ph-	NH_2_	10.0	2.38	>65	–	–
**17**	3-Py-	3-F-Ph-	NH_2_	12.2	3.51	>100	–	–
**18**	3-Py-	4-OMe-Ph-	NH_2_	14.6	1.98	>100	–	–
**19**	3-Py-	4-Br-Ph-	NH_2_	7.9	3.20	>100	–	–
**20**	3-Py-	3,4-OMe-	NH_2_	20.3	6.24	>100	–	–
**21**	3-Py-	3,4-O_2_CH_2_-Ph-	NH_2_	12.9	0.63	>100	–	–
**22**	2-Py-	2-OMe-Ph-	H	8.7	0.19	>15	–	–
**23**	2-Py-	2-OMe-Ph-	NH_2_	13.9	1.96	>100	–	–
**24**	4-Py-	2-OMe-Ph-	H	13.0	5.00	>100	–	–
**25**	4-Py-	2-OMe-Ph-	NH_2_	24.2	0.69	>70	–	–
**Melarsoprol**				0.011	0.005			
**Podophyllotoxin**						0.019	0.007	

#### 2.2.1 4-Phenyl-6-(pyridin-3-yl)pyrimidine derivatives

Derivative **1**, 4-phenyl-6-(pyridin-3-yl)pyrimidine, exhibited low micromolar antitrypanosomal activity with an IC_50_ value of 4.8 μM whilst showing negligible toxicity towards L6 cells (>100 μM). In comparison, the di-pyridyl substituted analogue **2** demonstrated less antitrypanosomal activity but similarly to **1** remained non-toxic to L6 cells. Compounds **3** and **4**, the 2-methoxyphenyl and 2-bromophenyl analogues both demonstrated antitrypanosomal IC_50_ values of 19.6 μM and 13.5 μM with no toxicity determined against L6 cells. The 3-methoxypheny analogue **5** showed a lower antitrypanosomal IC_50_ value of 8.0 μM, however, **6**, the 3-bromophenyl derivative exhibited an increased antitrypanosomal effect with an IC_50_ value of 2.0 μM. Subsequently, derivative **7**, bearing the 3-fluorophenyl substituent had reduced activity in comparison to both the 3-bromo and 3-methoxyphenyl analogues with an IC_50_ value of 12.4 μM but again no toxicity was observed against L6 cells. In comparison, **8** bearing a 4-methoxyphenyl substituent showed better antitrypanosomal activity than the other methoxyphenyl substituted analogues **3** and **5** with an IC_50_ value of 6.8 μM. The 3,4-dimethoxyphenyl substituted analogue **9** showed less activity than both **5** and **8** with similar cytotoxicity values observed and **10**, bearing a methylenedioxyphenyl substituent demonstrated an IC_50_ value of 6.7 μM.

#### 2.2.2 4-Phenyl-6-(pyridin-3-yl)pyrimidin-2-amine derivatives

In relation to **1**, derivative **11**, the 4-phenyl-6-(pyridin-3-yl)pyrimidin-2-amine, exhibited greater than 3-fold higher antitrypanosomal activity with an IC_50_ value of 13.8 μM and **12** also demonstrated less antitrypanosomal activity than its counterpart **2**. Compound **13**, bearing a 2-methoxyphenyl substituent, showed submicromolar antitrypanosomal activity with an IC_50_ value of 0.38 μM which was greater than 50-fold lower than that observed for **3**. Although **13** exhibited a CC_50_ value of 23 μM against L6 cells, this compound was over 60 times more selective against trypanosomes and provided a compound of interest from the 4-phenyl-6-(pyridin-3-yl)pyrimidin-2-amine library. Replacing the methoxy group with Br resulted in a reduction of antitrypanosomal activity as observed with **14** but did not exhibit cytotoxic results against L6 cells. By viewing **13** as a pharmacophore of interest, moving the methoxy group to the 3-position to give **15** showed less antitrypanosomal activity. The 3-bromophenyl analogue **16** demonstrated similar activity to **15** although its 4-phenyl-6-(pyridin-3-yl)pyrimidine counterpart **6** showed low micromolar antitrypanosomal activity. Replacing Br with F as shown with **17** did not increase antitrypanosomal potency and demonstrated a similar result to **7**. Derivative **18**, designed with a 4-methoxyphenyl substituent also showed comparable activity to **17** but the 4-bromophenyl substituted compound **19** exhibited nearly two times more activity than **18**. Incorporating a 3,4-dimethoxyphenyl group into the framework as shown with **20** raised the IC_50_ value to 20.3 μM and in line with **17**, **18** and **19** did not demonstrate cytotoxic activity. In comparison, **21** showed nearly two times less activity than **10** but again didn’t demonstrate cytotoxic activity. Furthermore, to add to our findings **22**, **23** and **24** were prepared bearing the 2-methoxyphenyl group for the B-ring but with either a 2- or 4- positioned N in the pyridyl A-ring. However, the results obtained did not improve on **13**, the 4-(2-methoxyphenyl)-6-(pyridin-3-yl)pyrimidin-2-amine, as the antitrypanosomal values were 8.7, 13.9, 13.0 and 24.2 μM for **22**, **23**, **24** and **25** respectively with no notable cytotoxicity recorded apart from compound **22** which exhibited a CC_50_ value of 17.0 μM.

**Fig. 3:**
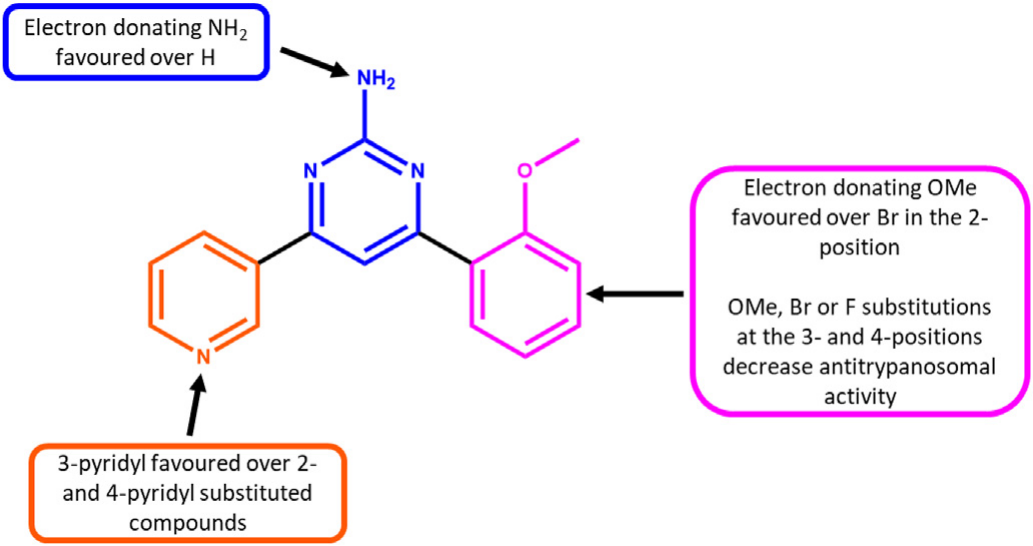
SAR observations for antitrypanosomal pyrimidines explored.

With the overall data observed, it can be seen that antitrypanosomal activity is favoured with a 3-pyridyl A-ring, and 2-methoxyphenyl B-ring and a 2-aminopyrimidine core (Fig. 3).

### 2.3 *In vitro* ADME-Tox and *In silico* ADME assessment

**Fig. 4:**
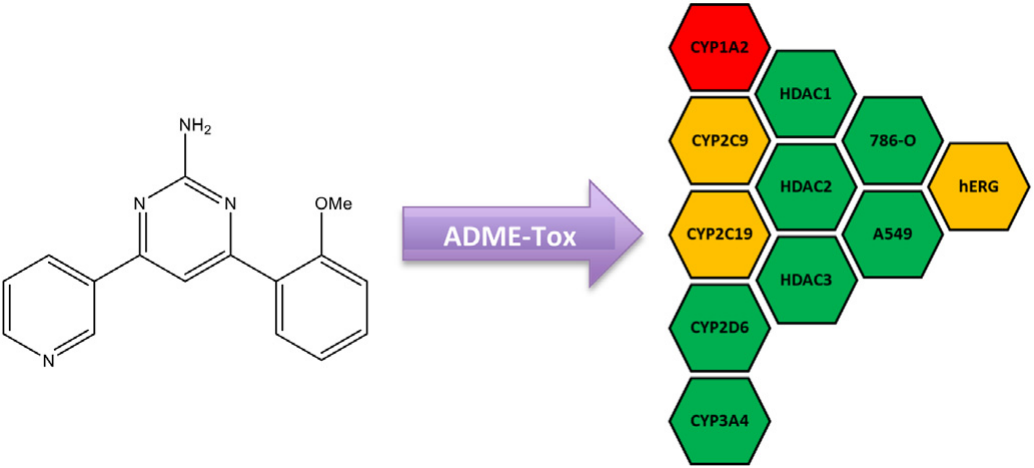
Data generated from the *in vitro* ADME-Tox assessment of compound **13**. The results were reported as percentage inhibition where 0–50% inhibition is indicated as a green tile, 51–70% as amber and 71–100% as a red tile.

Compound **13** was selected for further *in vitro* ADME-Tox studies based on the sub-micromolar antitrypanosomal activity observed and selectivity over L6 cells. This included screening Compound **13** at 10 μM against a panel of cytochrome P450s (CYP1A2, CYP2C9, CYP2C19, CYP2D6 and CYP3A4), histone deacetylases (HDAC1, HDAC3 and HDAC6) and hERG (Fig. 4). Cytotoxicity was also determined against 786-O and A549 cells.

Compound **13** showed favourable results against CYP3A4 and CYP2D6, some inhibition against CYP2C9 and CYP2C19 and inhibitory activity against CYP1A2. No inhibition was observed against HDAC 1, 3 and 6 and **13** was not cytotoxic towards 786-O and A549 cells. This dataset shows **13** to be a promising early stage pre-clinical compound for further development to increase on-target activity and decrease off-target toxicity. Compound **13** was further evaluated *in silico* using the ADME prediction program QikProp and the findings are reported below in Table 2 (Schrödinger Release 2020–3: Maestro; LigPrep; QikProp, Schrödinger, LLC, New York, NY, 2020). All properties calculated are satisfactory physico-chemical properties for compound **13**.

**Table 2:** QikProp ADME Predictions with ranges as described for the program: CNS, Predicted central nervous system activity on a −2 (inactive) to +2 (active) scale; QPlogPo/w, predicted octanol/water partition coefficient (−2.0–6.5); QPPCaco, Predicted apparent Caco-2 cell permeability in nm/sec (<25 poor, >500 great); QPlogBB, predicted brain/blood partition coefficient (−3.0–1.2); HOralAbs, predicted qualitative human oral absorption (1, 2, or 3 for low, medium, or high); Ro5, number of violations of Lipinski’s rule of five; and Ro3, number of violations of Jorgensen’s rule of three.

Compound	Predicted ADME Properties						
	CNS	QPlogPo/w	QPPCaco	QPlogBB	HOralAbs	Ro5	Ro3
**13**	−1	2.602	906.463	−0.722	3	0	0

### 2.4 *In silico* target exploration

Previous findings have shown pyrimidines to putatively inhibit cruzain, a cysteine protease expressed in *Trypanosoma cruzi* (*T. cruzi*) which is essential for parasite survival [17]. Based on this, we performed docking analyses of our pyrimidine library against rhodesain, an analogous cysteine protease essential for parasite survival since it is involved in several pathological processes in the host, including the crossing of the parasite through the blood brain barrier, turnover of variant surface glycoproteins (VSGs) and degradation of host immunoglobulins [18,19].

**Fig. 5:**
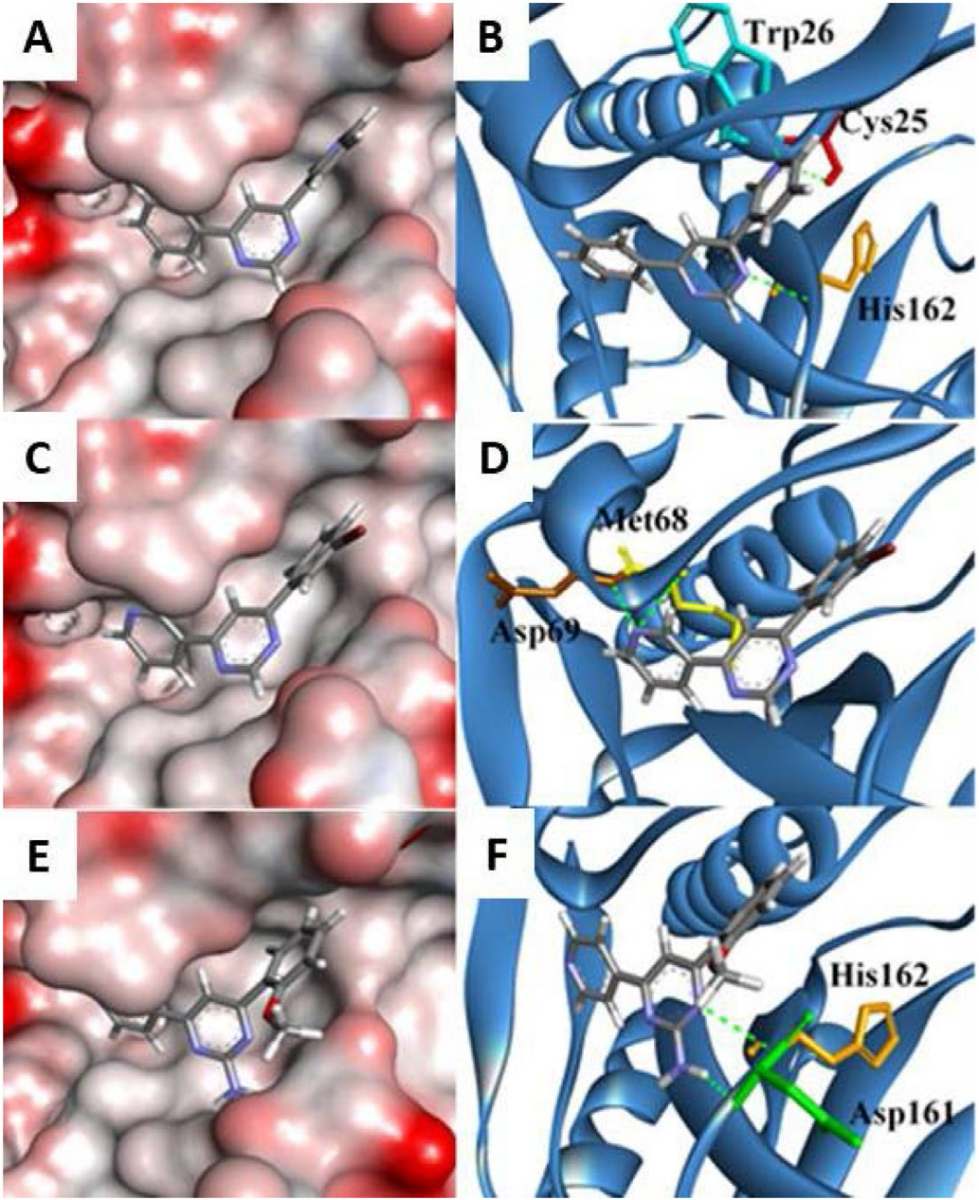
Docking of compounds **1**, **6** and **13** with rhodesain. Red and grey surfaces indicate partially negative and neutral charges, respectively. The protein folds were shown as blue ribbons. Hydrogen bonding amino acid residues were depicted as: Cys25 (red), Trp26 (turquoise), Met68 (yellow), Asp69 (brown), Asp161 (green) and His162 (orange).

The 4-phenyl-6-(pyridine-3-yl)pyrimidines docked into the binding site with similar binding modes and were predicted to occupy the S2 binding pocket of the catalytic site. Both the A and B rings of compounds can occupy the hydrophobic pocket deeply buried into the S2 site and can form favourable hydrophobic interactions with nearby hydrophobic residues: Ala136; Ile137; Ala138; Ala208; and Leu67. The importance of hydrophobic interactions between the mentioned residues with bioactive ligands has been documented [17,20,21]. The pyrimidine core can interact with nearby hydrophobic Leu160 and Leu67 residues and hydrogen bonds with His162. In this case, when the A ring is oriented towards the catalytic triad, the 3-pyridine nitrogen atom can form hydrogen bonds with Trp26 and catalytic Cys25 residues as seen for derivative **1** (Fig. 5A and B). In the hydrophobic pocket, the 3-pyridine nitrogen was able to form hydrogen bonds with the backbones of Met68 and Asp69 as seen for derivative **6** (Fig. 5C and D). The role of catalytic Cys25 and His162 residues are renowned for their catalytic function in cysteine proteases and intermolecular interactions with the residues implicates cysteine protease inhibitory effect [22,23]. The amino group at R^1^ was seen frequently to form a hydrogen bond with Asp161 residue as in the case of derivative **13** (Fig. 5E and F).

### 2.5 Nuclear magnetic resonance

#### 2.5.1 Saturation Transfer Difference (STD) and WaterLOGSY

**Fig. 6:**
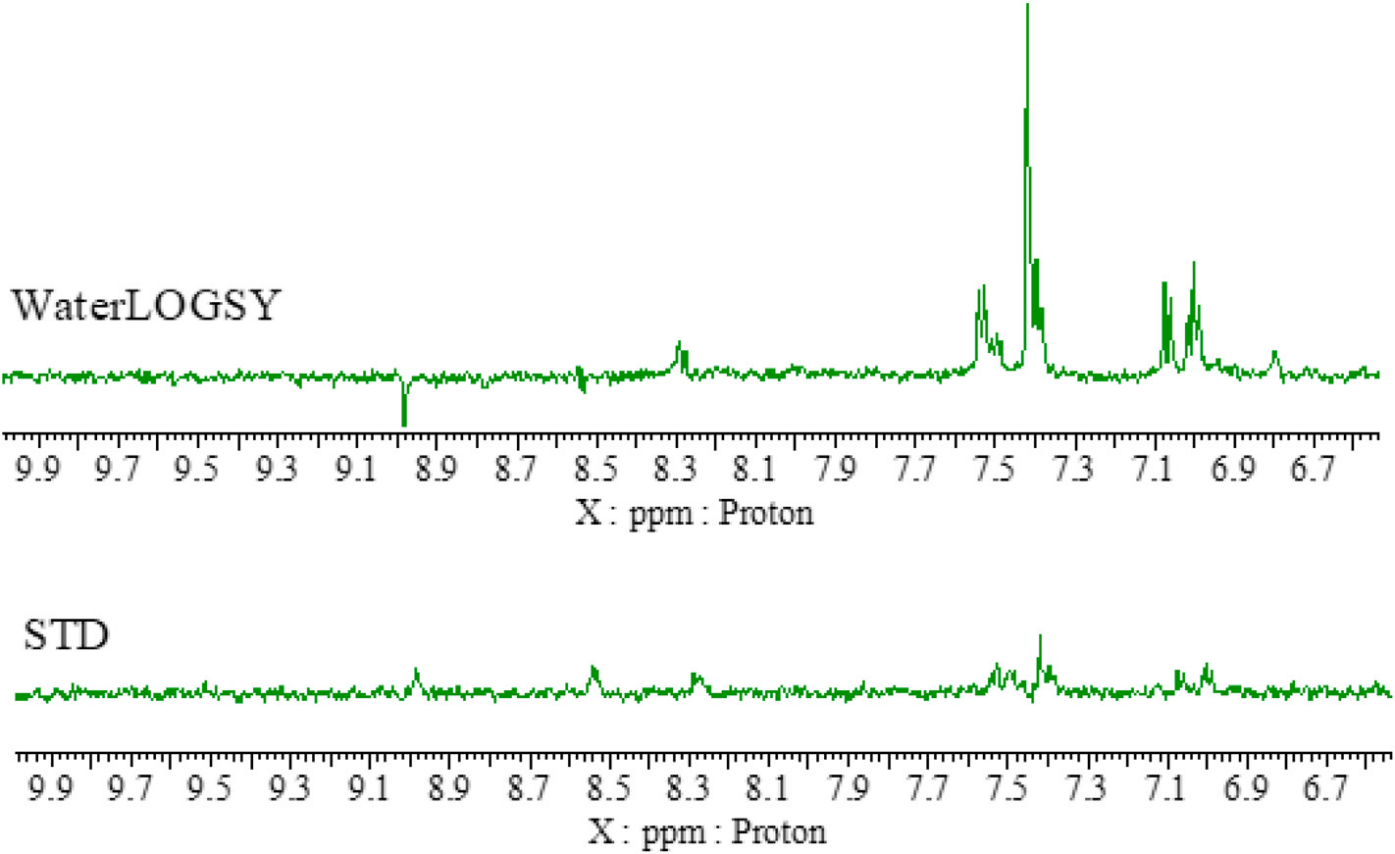
STD and WaterLOGSY spectra of compound 13 in the presence of rhodesain.

In order to support the outcome observed from docking studies, protein-ligand interactions were probed via Saturation Transfer Difference (STD) and WaterLOGSY experiments [24,25]. STD is an NMR technique where the protein is selectively saturated and, if the ligand is binding to the protein in the timescale of the experiment, the saturation gets transferred to the ligand via intermolecular NOE and only then it is possible to observe an NMR signal. WaterLOGSY relies on the detection of the intermolecular NOE between water (which is selectively irradiated) and the ligand. The sign of the NOE depends on the rotational correlation time of the molecule, thus large molecules such as proteins, have an opposite NOE sign than small molecules such as the pyrimidines investigated. If the pyrimidines bind to the protein, their rotational correlation time changes and the sign of the signals in the WaterLOGSY experiment will be opposite than in the absence of a binding protein. NMR signals for **13** were detected by STD, confirming binding to rhodesain (Fig. 6). The WaterLOGSY results, which as expected is generally a more sensitive technique, further corroborate our findings [26]. However, proton signals at 8.5 ppm and 9.0 ppm present in the STD results are almost absent or have the opposite sign in the WaterLOGSY spectra. The STD indicates these protons are binding to rhodesain, while the WaterLOGSY, as it is solvent-mediated, indicates that these protons have weak solvent accessibility meaning they are buried in the protein [27,28].

### 2.6 *In vitro* rhodesain activity

Based on the *in silico* docking studies and outcomes of the NMR experiments performed, compound **13** was selected for evaluation of its potential inhibition against rhodesain *in vitro*. Compound **13**, at a concentration of 50 μM was assayed with varying concentrations of the Cbz-Phe-Arg-AMC substrate (10, 5 and 1.25 μM) and a maximum of 15% inhibition of rhodesain enzymatic activity was detected. Although *in silico* docking and NMR experiments showed favourable protein-ligand interactions, *in vitro* enzymatic screening data indicates that the weak experimental inhibition observed signifies rhodesain is not the proposed target for compound **13**. This result warrants further investigations into the antitrypanosomal mechanism of action of compound **13** but also establishes a new chemical scaffold for further development with the potential of inhibiting rhodesain.

## 3 Conclusion

Our work has described the discovery of novel 4-phenyl-6-(pyridine-3-yl)pyrimidines demonstrating promising antitrypanosomal activities *in vitro*. In particular, compound **13** exhibited potent antitrypanosomal activity against *T.b.r* with an IC_50_ value of 0.38 μM. Limited off-target liabilities were recorded against CYP450s and HDACs and there was no notable hERG or cell cytotoxicity. To our knowledge, this is the first report detailing such findings encompassing this compound class in relation to *T.b.r*. *In silico* docking and *in vitro* NMR experiments indicated the cysteine protease rhodesain as a putative target for our novel compound library and subsequent *in vitro* enzymatic studies indicated compound **13** had a weak inhibitory effect. These findings suggest the antitrypanosomal effects observed for our pyrimidines are not linked to rhodesain inhibition, prompting further investigations to elucidate the mechanism of action. *In vivo* evaluation and further chemical development of the presented chemical scaffold from both a phenotypic and target-based drug design approach could exploit the antikinetoplastid potential of this compound class and we shall report further findings in due course.

## 4 Experimental

### 4.1 General

All solvents and chemicals were used as purchased without further purification. The ^1^H and ^13^C NMR spectra were recorded on a JEOL ECZ-R 600 MHz or Bruker Avance AV400 NMR spectrometer. Chemical shifts are reported in *δ* units (ppm) relative to either TMS or the residual solvent signal. IR spectra were recorded on a Bruker Alpha ATR IR instrument. HRMS was performed using a Thermo Scientific LTQ Orbitrap XL at the EPSRC UK National Mass Spectrometry Facility at Swansea University. Melting points were determined on Gallenkamp melting point apparatus. TLC was performed on Merck Silica Gel 60F254 coated plates and visualised under UV light (254/366 nm). Fisher silica gel 60 (20–45 or 35–70 μm) was used for flash chromatography.

### 4.2 Chemistry

#### 4.2.1 General method for synthesis of chalcones

Chalcones were synthesised using the methods described by our previous work [13].

#### 4.2.2 General method for the synthesis of 4-phenyl-6-(pyridine-3-yl)pyrimidines

In an inert atmosphere sodium ethoxide was generated *in situ* by reacting sodium metal (4 mmol) with dry EtOH (50 mL) under reflux. After the sodium metal had dissolved, either guandine hydrochloride (1.5 mmol) or formamidine hydrochloride (1.5 mmol) was added depending on the target pyrimidine. Subsequently, the corresponding chalcone (1.0 mmol) was added and the solution was left to stir (18 h) under reflux. The completion of the reaction was indicated by TLC analyses and the reaction was quenched with water (50 mL). The crude mixture was extracted with ethyl acetate (3 × 50 mL), washed with brine (50 mL) and dried over MgSO_4_. The crude product was purified by recrystallisation in ethanol or flash column chromatography (ethyl acetate/hexane or dichloromethane/methanol as the eluent).

##### 4.2.2.1 4-Phenyl-6-(pyridin-3-yl)pyrimidine (1)

Yield: 18%, mp: 116–118 °C. ^1^H NMR (400 MHz, CDCl_3_): *δ* 9.31 (1H, s), 8.73 (1H, d, *J* = 3), 8.71 (1H,s), 8.67 (1H, d, *J* = 5), 8.38–8.33 (2H, m), 7.61–7.54 (4H, m).^13^C NMR (101 MHz, CDCl_3_): *δ* 165.08, 162.32, 159.42, 151.72, 148.49, 136.61, 134.71, 132.73, 131.27, 129.15, 127.27, 123.83, 112.82. IR, υ_max_/cm^−1^: 3038, 1677, 1412, 1369, 1020. HRMS: *m/z* found 234.1028, C_15_H_12_N_3_ (M + H^+^) requires 234.1026.

##### 4.2.2.2 4,6-*Bis*(Pyridin-3-yl)pyrimidine (2)

Yield: 63%, mp: 206–207 °C. ^1^H NMR (400 MHz, DMSO‑*d*_6_): *δ* 9.54 (2H, s), 9.40 (1H, s), 8.84 (1H, s), 8.78 (2H, d), 8.71 (2H, dd, *J* = 5, 1), 7.61–7.65 (2H, m). ^13^C NMR (101 MHz, DMSO‑*d*_6_): *δ* 162.07, 159.13, 151.88, 148.56, 134.79, 131.73, 123.96, 113.48. IR, υ_max_/cm^−1^: 3038, 1591, 1571, 1414, 1371, 1259. HRMS: *m/z* found 235.0981, C_14_H_11_N_4_ (M + H^+^) requires 235.0978.

##### 4.2.2.3 4-(2-Methoxyphenyl)-6-(pyridin-3-yl)pyrimidine (3)

Yield: 21%, mp: 102–104 °C. ^1^H NMR (400 MHz, CDCl_3_): *δ* 9.37 (1H, s), 9.36 (1H, s), 8.77 (1H, bs), 8.57 (1H, bs), 8.56 (1H, s), 7.94 (1H, d, *J* = 5), 7.63–7.61 (1H, m), 7.55 (1H, t, *J* = 5), 7.23 (1H, d, *J* = 5), 7.13 (1H, t, *J* = 6), 3.90 (3H, s). ^13^C NMR (101 MHz, CDCl_3_): *δ* 163.76, 161.13, 159.08, 157.93, 151.42, 148.60, 134.72, 132.03, 131.10, 125.96, 123.77, 121.28, 117.71, 111.59, 55.73. IR, υ_max_/cm^−1^: 2922, 1589, 1574, 1460, 1251. HRMS: *m/z* found: 264.1131C_16_H_14_N_3_O (M + H^+^) requires 264.1131.

##### 4.2.2.4 4-(2-Bromophenyl)-6-(pyridin-3-yl)pyrimidine (4)

Yield: 12%, Yellow oil. ^1^H NMR (400 MHz, CDCl_3_): *δ* 9.40 (1H, d, *J* = 2), 9.34 (1H, d, *J* = 1), 8.77 (1H, dd, *J* = 5, 1), 8.63 (1H, dt, *J* = 8, 2), 8.45 (1H, d, *J* = 1), 7.83 (1H, dd, *J* = 8, 1), 7.70 (1H, dd, *J* = 8, 2), 7.57–7.63 (2H, m), 7.48 (1H, td, *J* = 8, 2). ^13^C NMR (100 MHz, DMSO‑*d*_6_): *δ* 166.18, 161.06, 158.70, 151.88, 149.70, 148.39, 147.26, 138.67, 134.73, 133.31, 131.45, 128.05, 124.03, 120.86, 117.67. IR, υ_max_/cm^−1^: 3054, 2924, 1497, 1451. HRMS: *m/z* found 312.0133, C_15_H_11_^79^BrN_3_ (M + H^+^) requires 312.0131.

##### 4.2.2.5 4-(3-Methoxyphenyl)-6-(pyridin-3-yl)pyrimidine (5)

Yield: 8%, mp: 124–125 °C. ^1^H NMR (400 MHz, DMSO‑*d*_6_): *δ* 9.53 (1H, d), 9.34 (1H, s), 8.79–8.77 (1H, m), 8.74 (1H, s), 8.72 (1H, dd, *J* = 5, 1),7.98 (1H, d, *J* = 6), 7.92 (1H, s), 7.66–7.61 (1H, m), 7.52, (1H, td, *J* = 5, 1), 7.16 (1H, dt, *J* = 5, 1), 3.89 (3H, s). ^13^C NMR (101 MHz, DMSO‑*d*_6_) *δ* 163.72, 161.88, 159.82, 158.93, 151.71, 148.54, 137.52, 134.76, 131.90, 130.08, 123.89, 119.70, 117.20, 113.11, 112.31, 55.35. IR, υ_max_/cm^−1^: 1594, 1571, 1523, 1495, 1296, 1268. HRMS: *m/z* found: 264.1134C_16_H_14_N_3_O(M + H^+^) requires 264.1131.

##### 4.2.2.6 4-(3-Bromophenyl)-6-(pyridin-3-yl)pyrimidine (6)

Yield: 22%, mp: 162–163 °C. NMR ^1^H (400 MHz, DMSO‑*d*_6_): *δ* 9.55 (1H, s), 9.37 (1H, s), 8.81 (1H, s), 8.79–8.77 (1H, m), 8.72 (1H, d, *J* = 2), 8.61 (1H, s), 8.41 (1H, d, *J* = 2), 7.81 (1H, d, *J* = 2), 7.60–7.57 (1H, m), 7.52 (1H, td, *J* = 6, 2). ^13^C NMR (101 MHz, DMSO‑*d*_6_): *δ* 162.32, 162.14, 158.98, 151.82, 148.58, 138.38, 134.78, 133.93, 131.75, 131.09, 129.87, 126.30, 123.89, 122.52, 113.29. IR, υ_max_/cm^−1^: 3056, 2919, 1583, 1561, 1518, 1460, 1240. HRMS: *m/z* found: 312.0314C_15_H_11_Br_79_N_3_ (M + H^+^) requires 312.0131.

##### 4.2.2.7 4-(3-Fluorophenyl)-6-(pyridin-3-yl)pyrimidine (7)

Yield: 14%, mp: 193–195 °C. NMR ^1^H (400 MHz, DMSO‑*d*_6_): *δ* 9.54 (1H, s), 9.37 (1H, s), 8.80 (1H, s), 8.78 (1H, d, *J* = 3), 8.71 (1H, d, *J* = 5), 8.32 (1H, d, *J* = 1), 8.27 (1H, d, *J* = 6), 8.24 (1H. d, *J* = 7), 7.68–7.61 (1H, m), 7.45 (1H, t, *J* = 5). ^13^C NMR (100 MHz, DMSO‑*d*_6_): *δ* 163.52 (d, *J* = 241), 163.14, 162.71, 159.59, 152.36, 149.13, 139.14 (d, *J* = 8), 135.36, 132.32, 131.62 (d, *J* = 8), 124,58, 123, 92, 118.69 (d, *J* = 23), 114.55 (d, *J* = 28), 113.92. IR, υ_max_/cm^−1^: 3051, 2921, 2852, 1585, 1571, 1448, 1261. HRMS: *m/z* found 252.0934, C_15_H_11_FN_3_ (M + H^+^) requires 252.0932.

##### 4.2.2.8 4-(4-Methoxyphenyl)-6-(pyridin-3-yl)pyrimidine (8)

Yield: 21%, mp: 165–167 °C. ^1^H NMR (400 MHz, DMSO‑*d*_6_): *δ* 9.52 (1H, s), 9.27 (1H, s), 8.77–8.75 (1H, m), 8.69 (1H, dd, *J* = 6, 1), 8.64 (1H, s), 8.37 (2H, mAA′), 7.64–7.60 (1H, m), 7.13 (2H, mBB’), 3.87 (3H, s). ^13^C NMR (101 MHz, DMSO‑*d*_6_): *δ* 163.54, 161.95, 161.45, 158.89, 151.59, 148.44, 134.65, 132.05, 129.03, 128.35, 123.87, 114.34, 111.96, 55.42. IR, υ_max_/cm^−1^: 1590, 1512.1467, 1372, 1247, 1174, 1022. HRMS: *m/z* found 264.1134, C_16_H_14_N_3_O (M + H^+^) requires 264.1131.

##### 4.2.2.9 4-(3,4-Dimethoxyphenyl)-6-(pyridin-3-yl)pyrimidine (9)

Yield: 18%, mp: 131–134 °C. ^1^H NMR (400 MHz, CDCl_3_): *δ* 9.53 (1H, s), 9.28 (1H, s), 8.77 (1H, d, *J* = 3), 8.69 (1H, dd, *J* = 5, 1), 8.67 (1H, s), 8.05 (1H, dt, *J* = 6, 1), 7.94 (1H, s), 7.64–7.61 (1H, m), 7.15 (1H, dd, *J* = 6, 1), 3.91 (3H, s), 3.87 (3H, s). ^13^C NMR (101 MHz, DMSO‑*d*_6_): *δ* 163.34, 161.21, 158.62, 151.45, 151.39, 149.04, 148.27, 134.46, 131.82, 128.15, 123.67, 120.50, 111.83, 111.40, 109.89, 55,72, 55.69. IR, υ_max_/cm^−1^: 2967, 2915, 2838, 1674, 1587, 1511, 1372, 1249. HRMS: *m/z* found 294.1236, C_17_H_16_N_3_O_2_ (M + H^+^) requires 294.1237.

##### 4.2.2.10 4-(2*H*-1,3-benzodioxol-5-yl)-6-(pyridin-3-yl)pyrimidine (10)

Yield: 15%, mp: 173–175 °C. ^1^H NMR (400 MHz, DMSO‑*d*_6_): *δ* 9.51 (1H, s), 9.26 (1H, s), 8.75 (1H, d, *J* = 3), 8.68 (1H, d, *J* = 5), 8.63 (1H, s), 8.02 (1H, d, *J* = 6), 7.96 (1H, s), 7.61 (1H, dd, *J* = 6, 5), 7.12 (1H, dd, *J* = 6, 1), 6.15 (2H, s). ^13^C NMR (101 MHz, DMSO‑*d*_6_) *δ* 163.29, 161.57, 158.81, 151.63, 150.07, 148.47, 148.18, 134.67, 131.97, 130.16, 123.86, 122.37, 112.20, 108.62, 107.10, 101.78. IR, υ_max_/cm^-1^:2920, 2852, 1678, 1585, 1524, 1502, 1425, 1252. HRMS: *m/z* found 278.0926, C_16_H_12_O_2_N_3_ (M + H^+^) requires 278.0924.

##### 4.2.2.11 4-Phenyl-6-(pyridin-3-yl)pyrimidin-2-amine (11)

Yield: 18%, mp: 172–175 °C. ^1^H NMR (400 MHz, DMSO‑*d*_6_): *δ* 9.40 (1H, s), 8.71 (1H, d, *J* = 3), 8.56 (1H, dd, *J* = 3), 8.28–8.23 (2H, m), 7.82 (1H, d, *J* = 2), 7.59–7.55 (1H, m), 7.55–7.51 (3H, m), 6.90 (2H, s). ^13^C NMR (101 MHz, DMSO‑*d*_6_): *δ* 165.13, 164.00, 162.78, 151.08, 148.24, 137.08, 134.36, 132.79, 130.58, 128.60, 127.02, 123.66, 102.08. IR, υ_max_/cm^−1^: 3320 (NH), 3155(NH), 1650, 1590, 1544, 1357. HRMS: *m/z* found 249.1137, C_15_H_13_N_4_ (M + H^+^) requires 249.1135.

##### 4.2.2.12 4,6-Bis(Pyridin-3-yl)pyrimidin-2-amine (12)

Yield: 20%, mp: 229–230 °C. ^1^H NMR (400 MHz, CDCl_3_): *δ* 9.41 (2H, s), 8.72 (2H, dt, *J* = 3, 1), 8.57 (2H, dd, *J* = 5, 2), 7.93 (2H, d, *J* = 1), 7.58 (2H, dd, *J* = 5, 3), 7.00 (2H, s). ^13^C NMR (101 MHz, CDCl_3_): *δ* 164.16, 163.69, 151.52, 148.57, 134.57, 132.98, 123,64, 103.93. IR, υ_max_/cm^−1^: 3340 (NH), 3220 (NH), 3064, 1645, 1583, 1549, 1365. HRMS: *m/z* found 250.1090, C_14_H_12_N_5_ (M + H^+^) requires 250.1087.

##### 4.2.2.13 4-(2-Methoxyphenyl)-6-(pyridin-3-yl)pyrimidin-2-amine (13)

Yield: 16%. mp: 108–110 °C. ^1^H H NMR (400 MHz, CDCl_3_): *δ* 9.22 (1H, d, *J* = 1), 8.69 (1H, dd, *J* = 5, 1), 8.34 (1H, dt, *J* = 8, 2), 7.86 (1H, dd, *J* = 8, 1), 7.64 (1H, s), 7.39–7.45 (2H, m), 7.09 (1H, td, *J* = 8, 1), 7.02 (1H, d, *J* = 8), 5.25 (2H, s), 3.90 (3H, s). ^13^C NMR (101 MHz, CDCl_3_): *δ* 165.41, 163.56, 162.49, 157.64, 151.03, 148.63, 134.67, 133.59, 131.42, 130.68, 126.84, 123.60, 121.05, 111.51, 108.92, 55.70. IR, υ_max_/cm^−1^: 3400–3200 (NH), 2927 2837, 1677, 1597, 1534, 1279 (C–H). HRMS: *m/z* found 279.1244, C_16_H_15_N_4_O (M + H^+^) requires 279.1240.

##### 4.2.2.14 4-(2-Bromophenyl)-6-(pyridin-3-yl)pyrimidin-2-amine (14)

Yield: 25%, mp: 149–152 °C. ^1^H NMR (400 MHz, DMSO‑*d*_6_): *δ* 9.28 (1H, s), 8.69 (1H, d, *J* = 3), 8.45 (1H, d, *J* = 5), 7.76 (1H, d, *J* = 5) 7.48–7.58 (3H, m), 7.35–7.44 (2H, m), 6.98 (2H, s). ^13^C NMR (400 MHz, DMSO‑*d*_6_): *δ* 167.51, 163.68, 161.63, 150.43, 147.37, 139.72, 135.22, 133.01, 130.80, 130.60, 128.62, 127.75, 124.13, 120.57, 106.30. IR, υ_max_/cm^−1^: 3294 (NH), 3069, 2922, 2852, 1657, 1593, 1543, 1351. HRMS: *m/z* found 327.0243, C_15_H_12_Br_79_N_4_ (M + H^+^) requires 327.0240.

##### 4.2.2.15 4-(3-Methoxyphenyl)-6-(pyridin-3-yl)pyrimidin-2-amine (15)

Yield: 26%, mp: 162–164 °C. NMR: ^1^H NMR (400 MHz, DMSO‑*d*_6_): *δ* 9.40 (1H, s), 8.71 (1H, d, *J* = 3), 8.56 (1H, dd, *J* = 5, 1), 7.83 (1H, d, *J* = 5), 7.81 (1H, d, *J* = 1), 7.79 (1H, bs), 7.56 (1H, dd, *J* = 5, 3), 7.44 (1H, td, *J* = 5, 1), 7.10 (1H, d, *J* = 6), 6.88 (2H, s), 3.86 (3H, s). ^13^C NMR (101 MHz, DMSO‑*d*_6_): *δ* 164.93, 163.93, 162.78, 159.57, 151.07, 148.27, 138.59, 134.41, 132.77, 129.68, 123.67, 119.43, 116.35, 112.13, 102.22, 55.27. IR, υ_max_/cm^−1^: 3431 (NH), 3304 (NH), 3178, 0.2921, 2852, 1634, 1564, 1538, 1451. HRMS: *m/z* found 279.1243, C_16_H_15_N_4_O (M + H^+^) requires 279.1240.

##### 4.2.2.16 4-(3-Bromophenyl)-6-(pyridin-3-yl)pyrimidin-2-amine (16)

Yield: 45%, mp: 169–171 °C NMR: ^1^H NMR (400 MHz, DMSO‑*d*_6_): *δ* 9.41 (1H, s), 8.71 (1H, d, *J* = 3), 8.56 (1H, dd, *J* = 6, 1), 8.47 (1H, d, *J* = 1), 8.27 (1H, d, *J* = 5), 7.90 (1H, d, *J* = 1), 7.73 (1H, d, *J* = 5), 7.57 (1H, dd, *J* = 5, 3), 7.50 (1H, td, *J* = 6, 1) 6.97 (2H, s). ^13^C NMR (101 MHz, DMSO‑*d*_6_): *δ* 163.95, 163.39, 163.18, 151.20, 148.34, 139.39, 134.41, 133.22, 132.61, 130.78, 129.56, 126.00, 123.64, 122.22, 102.17. IR, υ_max_/cm^−1^: 3482 (NH), 3316 (NH), 3181, 2922, 2851, 1630, 1580, 1561.1461, 1248. HRMS: *m/z* found 327.0243, C_15_H_12_Br_79_N_4_ (M + H^+^) requires 327.0240.

##### 4.2.2.17 4-(3-Fluorophenyl)-6-(pyridin-3-yl)pyrimidin-2-amine (17)

Yield: 15%, mp: 196–198 °C. ^1^H NMR (400 MHz, DMSO‑*d*_6_): *δ* 9.41 (1H, s), 8.72 (1H, d, *J* = 3), 8.56 (1H, dd, *J* = 5, 2), 8.12 (1H, d, *J* = 6), 8.08 (1H, d, *J* = 7), 7.89 (1H, s), 7.62–7.54 (2H, m), 7.37 (1H, t, *J* = 6), 6.90 (2H, s). ^13^C NMR (101 MHz, DMSO‑*d*_6_): *δ* 165.24, 164.04, 163.63, 163.23 (d, *J* = 245), 151.43, 148.56, 139.69 (d, *J* = 8), 134.39, 133.13, 130.38 (d, *J* = 8), 123.60, 122.70 (d, *J* = 3), 117.62 (d, *J* = 22), 114.16 (d, *J* = 23), 104.04. IR, υ_max_/cm^−1^: 3483 (NH), 3319 (NH), 2921, 2852, 1634, 1584, 1569, 1447, 1351, 1261. HRMS: *m/z* found 267.1043, C_15_H_12_FN_4_ (M + H^+^) requires 267.1041.

##### 4.2.2.18 4-(4-Methoxyphenyl)-6-(pyridin-3-yl)pyrimidin-2-amine (18)

Yield: 27%, mp: 163–165 °C. ^1^H NMR (400 MHz, DMSO‑*d*_6_): *δ* 9.38 (1H, s), 8.70 (1H, d, *J* = 3), 8.53 (1H, dd, *J* = 6, 1), 8.23 (2H, d, *J* = 5), 7.76 (1H, s), 7.55 (1H, dd, *J* = 5, 3), 7.07 (2H, d, *J* = 5), 6.79 (2H, s), 3.84 (3H, s). ^13^C NMR (101 MHz, DMSO‑*d*_6_): *δ* 164.65, 163.89, 162.41, 161.34, 150.97, 148.19, 134.32, 132.91, 129.33, 128.63, 123.65, 113.93, 101.27, 55.31. IR, υ_max_/cm^−1^ 3326 (NH), 3186 (NH), 2921, 1642, 1580, 1451, 1359. HRMS: *m/z* found 279.1242, C_16_H_15_N_4_O (M + H^+^) requires 279.1240.

##### 4.2.2.19 4-(4-Bromophenyl)-6-(pyridin-3-yl)pyrimidin-2-amine (19)

Yield: 32%, mp: 210–211 °C. ^1^H NMR (400 MHz, DMSO‑*d*_6_): *δ* 9.38 (1H, s), 8.72–8.71 (1H, m), 8.54 (1H, d, *J* = 5), 8.20 (2H, d, *J* = 6), 7.84 (1H, s), 7.74 (2H, d, *J* = 6), 7.57–7.53 (1H, m), 6.92 (2H, s). ^13^C NMR (101 MHz, DMSO‑*d*_6_): *δ* 163.97, 163.92, 163.06, 151.17, 148.28, 136.27, 134.36, 132.66, 131.60, 129.04, 124.26, 123.66, 101.94. IR, υ_max_/cm^−1^: 34,854 (NH), 3323 (NH), 3072, 2921, 1627, 1592, 1536, 1455, 1360. HRMS: *m/z* found 327.0243, C_15_H_12_Br_79_N_4_ (M + H^+^) requires 327.0240.

##### 4.2.2.20 4-(3,4-Dimethoxyphenyl)-6-(pyridin-3-yl)pyrimidin-2-amine (20)

Yield: 24%, mp: 165–167 °C. ^1^H NMR (400 MHz, DMSO‑*d*_6_): *δ* 9.40 (1H, s), 8.70 (1H, d, *J* = 3), 8.54 (1H, dd, *J* = 5, 1), 7.88 (1H, dt, *J* = 6, 1), 7.81 (1H, s), 7.79 (1H, s), 7.56 (1H, dd, *J* = 5, 3), 7.09 (1H, dd, *J* = 6, 1), 6.80 (2H, s), 3.88 (3H, s), 3.84 (3H, s). ^13^C NMR (101 MHz, DMSO‑*d*_6_): *δ* 165.30, 164.78, 163.84, 162.40, 151.07, 148.72, 134.20, 134.32, 132.92, 129.53, 123.62, 120.31, 116.90, 112.75, 102.75, 55.66, 55.57. IR, υ_max_/cm^−1^: 3508 (NH), 3372 (NH), 2937, 1607, 1564, 1511, 1448, 1362. HRMS: *m/z* found 309.1347, C_17_H_17_N_4_O_2_ (M + H^+^) requires 309.1346.

##### 4.2.2.21 4-(2*H*-1,3-benzodioxol-5-yl)-6-(pyridin-3-yl)pyrimidin-2-amine (21)

Yield: 16%, mp: 236–238 °C. ^1^H NMR (400 MHz, DMSO‑*d*_6_): *δ* 9.38 (1H, s), 8.69 (1H, d, *J* = 3) 8.53 (1H, dd, *J* = 5, 1) 7.87 (1H, dt, *J* = 6, 1) 7.82 (1H, s) 7.75 (1H, s) 7.54 (1H, dd, *J* = 5, 3) 7.06 (1H, d, *J* = 5) 6.80 (2H, s), 6.12 (2H, s). ^13^C NMR (101 MHz, DMSO‑*d*_6_): *δ* 164.38, 163.82, 162.57, 151.01, 149.39, 148.25, 147.84, 134.31, 132.83, 131.23, 123.61, 121.73, 108.26, 106.95, 102.10, 101.54. IR, υ_max_/cm^−1^: 3492 (NH), 3309 (NH), 3195, 2900, 1633, 1571, 1504, 1370, 1253. HRMS: *m/z* found 293.1035, C_16_H_13_O_2_N_4_ (M + H^+^) requires 293.1033.

##### 4.2.2.22 4-(2-Methoxyphenyl)-6-(pyridin-2-yl)pyrimidine (22)

Yield: 12%, mp: 94–96 °C ^1^H NMR (400 MHz, CDCl_3_): *δ* 8.74 (1H, dd, *J* = 4, 1), 8.33 (1H, s), 8.31 (1H, s), 8.18 (1H, dt, *J* = 8, 1), 7.86 (1H, td, *J* = 8, 2), 7.79 (1H, dd, *J* = 8, 2), 7.47 (1H, ddd, *J* = 6, 5, 1), 7.37 (1H, ddd, *J* = 8, 7, 2), 6.99 (1H, td, *J* = 7, 1), 6.93 (1H, dd *J* = 8, 1), 3.92 (3H, s). ^13^C NMR (101 MHz, CDCl_3_): *δ* 158.92, 157.37, 154.61, 153.37, 148.84, 148.42, 139.97, 131.88, 128.84, 126.66, 124.25, 122.91, 121.16, 120.67, 111.20, 55.89. IR, υ_max_/cm^−1^: 2937, 2835, 1687.1600, 1584, 1492, 1435. HRMS: *m/z* found: 264.1134, C_16_H_14_N_3_O (M + H^+^) requires 264.1131.

##### 4.2.2.23 4-(2-Methoxyphenyl)-6-(pyridin-2-yl)pyrimidin-2-amine (23)

Yield: 19%, mp: 178–180 °C ^1^H NMR (400 MHz, CDCl_3_): *δ* 8.72 (1H, d, *J* = 3), 8.34 (1H, d, *J* = 6), 8.01 (1H, s), 7.99 (1H, td, *J* = 5, 1), 7.81 (1H, dd, *J* = 5, 1), 7.52 (1H, ddd, *J* = 5, 4, 1), 7.48–7.43 (1H, m), 7.18 (1H, d, *J* = 6), 7.08 (1H, t, *J* = 5), 6.73 (2H, s), 3.87 (3H, s). ^13^C NMR (101 MHz, CDCl_3_): *δ* 165.96, 163.72, 163.36, 157.64, 155.07, 149.48, 136.89, 131.09, 130.63, 127.37, 124.77, 121.75, 120.84, 111.39, 109.29, 55.73. IR, υ_max_/cm^−1^: 3293 (NH), 3172 (NH), 3003, 2964, 2931, 1627.1604, 1535, 1435. HRMS: *m/z* found: 279.1242C_16_H_15_N_4_O (M + H^+^) requires 279.1240.

##### 4.2.2.24 4-(2-Methoxyphenyl)-6-(pyridin-4-yl)pyrimidine (24)

Yield: 7%, mp: 124–126 °C. ^1^H NMR (400 MHz, CDCl_3_): *δ* 9.38 (1H, s), 8.81 (2H, bd, *J* = 2), 8.43 (1H, d, *J* = 1), 8.06 (1H, dd, *J* = 6, 1), 7.99 (2H, d, *J* = 4), 7.52–7.49 (1H, m), 7.15 (1H, td, *J* = 5, 1), 7.07 (1H, d, *J* = 6), 3.96 (3H, s). ^13^C NMR (101 MHz, CDCl_3_): *δ* 164.26, 161.07, 159.16, 157.90, 150.72, 144.71, 132.22, 131.13, 125.81, 121.36, 121.23, 118.15, 111.59.55.79. IR, υ_max_/cm^−1^: 2982, 1686, 1601, 1584, 1493, 1285. HRMS: *m/z* found: 264.1135C_16_H_14_N_3_O (M + H^+^) requires 264.1131.

##### 4.2.2.25 4-(2-Methoxyphenyl)-6-(pyridin-4-yl)pyrimidin-2-amine (25)

Yield: 9%, mp: 179–180 °C ^1^H NMR (400 MHz, CDCl_3_): *δ* 8.74 (2H, d, *J* = 4), 7.99 (2H, d, *J* = 4), 7.81 (1H, dd, *J* = 6, 1), 7.63 (1H, s), 7.47 (1H, td, *J* = 6, 1), 7.18 (1H, d, *J* = 6), 7.08 (1H, t, *J* = 5), 6.84 (2H, s), 3.89 (3H, s). ^13^C NMR (101 MHz, CDCl_3_): *δ* 165.86, 163.55, 162.47, 157.66, 150.48, 145.32, 131.55, 130.70, 126.73, 121.25, 121.11, 111.54, 109.27, 55.74. IR, υ_max_/cm^−1^: 3307 (NH), 3133 (NH), 1649, 1568, 1524, 1454, 1357. MS: *m/z* found: 279.1244C_16_H_15_N_4_O (M + H^+^) requires 279.1240.

### 4.3 Biological assays

#### 4.3.1 Antitrypanosomal and L6 cytotoxicity assays

Assays were performed as described by Bernal et al. [16]. *In vitro* antitrypanosomal activities of compounds were determined against *Trypanosoma brucei rhodesiense* STIB900 (bloodstream trypomastigotes) where the parasitic stock was isolated in 1982 from a human patient in Tanzania. Minimum Essential Medium (50 μl) supplemented with 25 mM HEPES, 1 g/L glucose, 1% MEM non-essential amino acids (100 ×), 0.2 mM 2-mercaptoethanol, 1 mM Na-pyruvate and 15% heat inactivated horse serum was added to each well of a 96-well microtiter plate. Serial drug dilutions from 100 to 0.002 μg/mL were prepared and 4 × 10^3^ bloodstream forms of *T. b. rhodesiense* STIB 900 in 50 μl was added to each well and the plate incubated at 37 °C under a 5% CO_2_ atmosphere for 70 h. Then, 10 μl of Alamar Blue (resazurin, 12.5 mg in 100 mL double-distilled water) was added to each well and incubation continued for a further 2–4 h. Plates were read with a Spectramax Gemini XS microplate fluorometer (Molecular Devices Cooperation, Sunnyvale, CA, USA) using an excitation wavelength of 536 nm and an emission wavelength of 588 nm. The data was analysed with SoftmaxPro (Molecular Devices Cooperation, Sunnyvale, CA, USA), which calculated IC_50_ values by linear regression and 4-parameter logistic regression from the sigmoidal dose inhibition curves.

*In vitro* cytotoxicity with L6 cells (a primary cell line derived from rat skeletal myoblasts) were performed in 96-well microtiter plates with each well containing 100 μl of RPMI 1640 medium supplemented with 1% l-glutamine (200 mM) and 10% fetal bovine serum and 4000 L6 cells. Serial drug dilutions from 100 to 0.002 μg/mL were prepared and after 70 h of incubation the plates were microscopically inspected to ensure the validity of control wells and sterile conditions. Then, 10 μl of Alamar Blue was added to each well and the plates incubated for an additional 2 h. Plates were read with a Spectramax Gemini XS microplate fluorometer (Molecular Devices Cooperation, Sunnyvale, CA, USA) using an excitation wave length of 536 nm and an emission wave length of 588 nm. The IC_50_ values were calculated by linear regression and 4-parameter logistic regression from the sigmoidal dose inhibition curves using SoftmaxPro software (Molecular Devices Cooperation, Sunnyvale, CA, USA).

#### 4.3.2 Pre-clinical *In vitro* ADME-Tox assays

Experiments for determining pre-clinical *in vitro* ADME-Tox activities were performed as described by Moraes et al. [29] and also described in the following sections 4.3.2.1 to **4.3.2.4**.

##### 4.3.2.1 Cytochrome (CYP) P450 inhibition assay

The luminescence based P450-Glo™ (Promega Corp.) assay was used in 384-well assay format.

The cytochrome P450 (CYP) panel included microsomal preparations of cytochromes P450 1A2, 2C9, 2C19, 2D6, and 3A4 (Corning) from baculovirus infected insect cells (BTI-TN-5B1−4) which express cytochromes P450 and cytochrome *c* reductase (and cytochrome *b*5 for 3A4). Compounds were added (100 nL/well in 1% DMSO v/v) using the Echo 550® Liquid Handler followed by addition of 5 μL/well of CYP450/substrate mixture. Following incubation for 30 min at 37 °C, the reaction was initiated by the addition of 5 μL/well of the NADPH regeneration mixture. By the end of a further 30 min incubation (37 °C), the CYP450 reaction was stopped and the luciferase reaction was initiated by the addition of 10 μL/well of the luciferin detection reagent, followed by an additional 30 min of incubation at 37 °C. The luminescence signal was detected using an Infinite® M1000 PRO plate reader. The negative controls yielded 0% inhibition (1% v/v DMSO) and standard CYP450 specific inhibitors were used as positive controls with 100% inhibition (CYP450 1A2, alpha-naphthoflavone; CYP450 2C9, sulfaphenazole; CYP450 2C19, troglitazone; CYP450 2D6, quinidine; CYP450 3A4, ketoconazole). The raw data were normalised relative to the positive and negative controls yielding the % inhibition for each compound.

##### 4.3.2.2 Histone deacetylase (HDAC) assay

Inhibition of histone deacetylase (HDAC) enzymes was determined using the bioluminogenic HDAC-Glo™ I/II assay (Promega Corp.) in 384-well assay format. Human recombinant HDAC enzymes were purchased from BPS Bioscience (San Diego, USA) and the standard inhibitor trichostatin A (Sigma-Aldrich) was dissolved to a yield stock solution in 100% v/v DMSO (stored at −20 °C). Plate handling was performed using a Cell Explorer HTS platform equipped with an Echo 550® Liquid Handler and Multidrop liquid handling system with luminescence measurements taken using an EnVision® multilabel 2103 reader. Compounds were added to plates (100 nL/well; 1% v/v DMSO) and the HDAC-Glo™ I/II assay reagent was prepared by (i) rehydration of lyophilised HDAC-Glo™ I/II substrate (with an acetylated peptide concentration of 100 μM) in 10 mL HDAC-Glo™ I/II assay buffer and (ii) addition of 10 μL of developer reagent (containing trypsin). The microtiter plates were mixed briefly by orbital shaking (500–700 rpm) and luminescence was measured at steady-state signal:background which was achieved after 20 min. The raw data were normalised relative to the positive and negative controls yielding the % inhibition for each compound.

##### 4.3.2.3 Cytotoxicity assays

A549 and 786-O cells were grown on surface-modified T175 cell culture flasks in Dulbecco’s Modified Eagle Medium with 10% fetal calf serum, streptomycin (100 μg/mL), and 100 U/mL penicillin G. At about 80% confluency, cells were washed, trypsinised, resuspended and counted in RPMI-1640 medium and seeded into 384-well microtiter plates (20 μL) at 500 cells/well. After 24 h of incubating at 37 °C in the presence of 5% CO2, test compounds were added to cells using an Echo 550 Liquid Handler and read after 48 h of incubation, the luminescence signal was read following addition of 20 μL using the CellTiter-GloCTG reagent. The raw data were normalised to percentage of cell growth by using the baseline growth and the corresponding NC containing only 1% v/v DMSO. The luminescence signal of each sample (S) was converted into percentage of cell growth compared with the average signal of the baseline control (BC). The raw data were normalised relative to the positive and negative controls yielding the % cytotoxicity for each compound.

##### 4.3.2.4 hERG cardiotoxicity assay

The Invitrogen Predictor™ hERG Fluorescence Polarisation Assay was used in 384-well assay format. To each well, 100 nL of the test and control compounds, 5 μL of homogenised membrane solution (undiluted) and 5 μL of the tracer (1 nM final concentration in assay) were added. The plates were incubated for 2 h at 25 °C in a humidity-controlled incubator and the fluorescence polarisation was measured using an EnVision® multilabel 2103 reader. The negative controls (0% inhibition) and positive controls with E−4031, a blocker of hERG-type potassium channels (100% inhibition) were used to normalise the raw data. The raw data were normalised relative to the positive and negative controls yielding the % cardiotoxicity for each compound.

##### 4.3.2.5 *In silico* ADME predictions

*In silico* ADME predictions were calculated using Schrodinger software (Schrödinger Release 2020–3: Maestro; LigPrep; QikProp, Schrödinger, LLC, New York, NY, 2020). The ligand was prepared using LigPrep and evaluated using QikProp in normal processing mode. Compound ADME Prediction ranges are as described for the program: CNS, Predicted central nervous system activity on a −2 (inactive) to +2 (active) scale; QPlogPo/w, predicted octanol/water partition coefficient (−2.0–6.5); QPPCaco, Predicted apparent Caco-2 cell permeability in nm/sec (<25 poor, >500 great); QPlogBB, predicted brain/blood partition coefficient (−3.0–1.2); HOralAbs, predicted qualitative human oral absorption (1, 2, or 3 for low, medium, or high); Ro5, number of violations of Lipinski’s rule of five; and Ro3, number of violations of Jorgensen’s rule of three.

### 4.4 Docking studies

The protein structure of *T.b.r* rhodesain was obtained from the Protein Data Bank (PDB) and the PDB ID is 2P7U [22,30]. The crystal structure was viewed using Discovery Studio visualizer version 3.5. All ligands including All ligands including water and co-crystallised ligands were removed and the proteins were protonated using the add hydrogen feature in Discovery Studio. The Genetic Optimisation for Ligand Docking (GOLD) version 5.8.1 was used as the docking engine and the GoldScore (GS) scoring function was implemented to validate the predicted binding modes and relative energies of the ligands [31,32]. The ligand structures were prepared in 3D format using the Scigress software package version 3.3.2 [33]. The ligands were energy minimised using the MM2 force-field followed by the semi-empirical PM6 method [34,35]. The centre of the docking grid was defined from the N2 amide bond of the co-crystallised ligand (x = −7.134, y = 1.467, z = 9.526) with 10 Å radius. Fifty docking runs were allowed for each ligand with default search efficiency (100%). The basic amino acids lysine and arginine were defined as protonated. Furthermore, aspartic and glutamic acids were assumed to be deprotonated. The co-crystallised ligand was removed and re-docked into the binding site and the root-mean squared deviation (RMSD) of heavy atoms measured by superimposing the top ranking re-docked conformation and the X-ray co-crystallised ligand using the GS scoring function (see supplementary information). The good overlays and low RMSD value of 2.45 Å was calculated suggesting the reliability and reproducibility of the docking protocol.

### 4.5 NMR experimental

NMR samples were prepared in a 50% DMSO‑*d*_6_ and 50% buffer solution (50 mM sodium acetate, 200 mM sodium chloride, 5 mM EDTA, 5% DMSO and 2 mM DTT). The overall concentration of rhodesain used was 50 μM and the final concentration of **13** was 5 mM.

All NMR studies were performed at 25 °C on a JEOL ECZ-R 600 MHz spectrometer equipped with a ROYAL room temperature probe. All experiments were run under automation, with the centre of the spectrum being automatically set via a script looking for the frequency of the tallest signal of a proton experiment preceding the actual experiment. The spectra were processed and analysed using JEOL Delta software. Saturation Transfer Difference experiments were performed with a saturation time of 6 s using REBURP pulses with a field strength of 92.3 Hz, with an off-resonance frequency of −200 ppm and an on-resonance frequency of 0.25 ppm in alternating 256 scans. A W5 Watergate solvent suppression scheme was used before acquisition to suppress the solvent signal. WaterLOGSY experiments were performed with a relaxation delay of 6 s and a mixing time of 1.6 s 256 scans were acquired and excitation sculpting was used before acquisition to supress the solvent signal. The resulting spectrum was phased using the same phase correction used in the experiment run in the sample without protein.

### 4.6 Rhodesain experimental

Enzyme assays with rhodesain were performed as described previously [[36], [37], [38]]. The assay buffer consisted of 50 mM sodium acetate (pH 5.5), 5 mM EDTA, 200 mM NaCl and 0.005% Brij35. The enzyme buffer consisted of 50 mM sodium acetate (pH 5.5), 5 mM EDTA, 200 mM NaCl and 2 mM DTT. The substrate Cbz-Phe-Arg-AMC (Bachem, purchased as HCl salt) was diluted from a 1 mM stock solution to reach concentrations of 10, 5 and 1.25 μM. The assay mixtures had a total volume of 200 μL and consisted of 180 μL assay buffer, 5 μL enzyme, 10 μL Me_2_SO (as negative control) or inhibitor and 5 μL of the substrate solution. Inhibition against rhodesain was measured using Cbz-Phe-Arg-AMC as the substrate, which releases AMC (7-amino-4-methylcoumarin) after amide bond cleavage by the enzyme. Proteolytic activity of the enzyme was monitored spectrophotometrically by the increase of fluorescence intensity by release of AMC (emission at 460 nm) upon hydrolysis.

### 4.7 X-ray crystallography

The crystal data was collected on a Bruker APEX 2000 CCD diffractometer using graphite monochromated Mo-Ka radiation (l = 0.71073 Å). The data were corrected for Lorentz and polarisation effects, and empirical absorption corrections were applied. The structures were solved by direct methods and refined by full-matrix least squares cycles on F2 for all data, using SHELX 2018/3 [39]. All hydrogen atoms were included in calculated positions (C–H = 0.95–0.98 Å) riding on the bonded atom with isotropic displacement parameters set to 1.5 Ueq(C) for methyl H atoms and 1.2 Ueq(C) for all other H atoms. All non-hydrogen atoms were refined with anisotropic displacement parameters. Crystallographic data for the structure reported in this paper has been deposited with The Cambridge Crystallographic Data Centre and allocated the deposition numbers CCDC: 2012793. Copy of the data can be obtained free of charge from The Cambridge Crystallographic Data Centre via www.ccdc.cam.ac.uk/data_request/cif.

## Author contributions

The manuscript was written through contributions of all authors ^a-k^. All authors have given approval to the final version of the manuscript and have contributed equally.

## Declaration of competing interest

The authors declare that they have no known competing financial interests or personal relationships that could have appeared to influence the work reported in this paper.
