# Supplementary material for: The discovery of novel antitrypanosomal 4-phenyl-6-(pyridin-3-yl)pyrimidines
Source: Eur J Med Chem. 2021 Jan 1;209:112871. doi: 10.1016/j.ejmech.2020.112871 (PMC7762786; doi:10.1016/j.ejmech.2020.112871)
Supplement: Multimedia component 1 [file mmc1.docx]

**Supplementary Information**

**The Discovery of Novel Antitrypanosomal 4-phenyl-6-(pyridin-3-yl)pyrimidines**

**
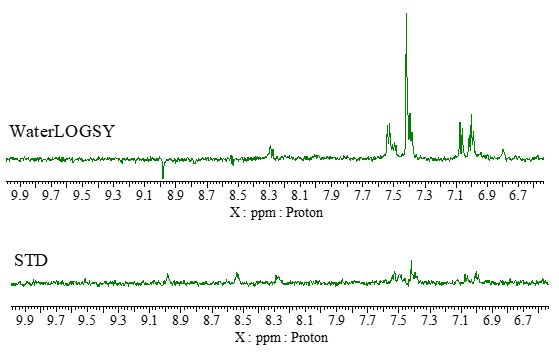
**WaterLOGSY and STD spectra of compound **13** and rhodesain.
